# Memristive Sisyphus circuit for clock signal generation

**DOI:** 10.1038/srep26155

**Published:** 2016-05-20

**Authors:** Yuriy V. Pershin, Sergey N. Shevchenko, Franco Nori

**Affiliations:** 1 Department of Physics and Astronomy and Smart State Center for Experimental Nanoscale Physics, University of South Carolina, Columbia, South Carolina 29208, USA; 2 CEMS, RIKEN, Saitama 351-0198, Japan; 3 Nikolaev Institute of Inorganic Chemistry SB RAS, Novosibirsk 630090, Russia; 4 B. Verkin Institute for Low Temperature Physics and Engineering, Kharkov 61103, Ukraine; 5 V. Karazin Kharkov National University, Kharkov 61022, Ukraine; 6 Physics Department, University of Michigan, Ann Arbor, Michigan 48109-1040, USA

## Abstract

Frequency generators are widely used in electronics. Here, we report the design and experimental realization of a memristive frequency generator employing a unique combination of only digital logic gates, a single-supply voltage and a realistic thresholdtype memristive device. In our circuit, the oscillator frequency and duty cycle are defined by the switching characteristics of the memristive device and external resistors. We demonstrate the circuit operation both experimentally, using a memristor emulator, and theoretically, using a model memristive device with threshold. Importantly, nanoscale realizations of memristive devices offer small-size alternatives to conventional quartz-based oscillators. In addition, the suggested approach can be used for mimicking some cyclic (Sisyphus) processes in nature, such as “dripping ants” or drops from leaky faucets.

Cyclic evolutions and relaxation oscillators are ubiquitous in nature. One example of these is the leaky faucet [Fig. 1(a)], where the suspended fluid mass increases gradually, until it suddenly decreases, when the droplet tears of f^1^,^2^,^3^. Other examples of relaxational dynamics can also be found in granular media^4^ as well as in mechanical^5^ and superconducting systems^6^. Similar dynamics can be even found in wildlife. For example, some types of ants^7^,^8^ can continuously climb a rod, aggregate, and eventually drop, right after a critical mass is accumulated [see Fig. 1(b)].

Analogous phenomena in driven dissipative systems are known as Sisyphus processes. According to Greek mythology, King Sisyphus was doomed to repeatedly push a rock uphill, which would then roll back down [Fig. 1(c)]. Recently, Sisyphus processes^9^ were studied in electric circuits based on superconducting^10^,^11^,^12^,^13^ and normal-state^14^,^15^,^16^ systems. In these circuits, a driven artificial atom, a qubit, was coupled to either a mechanical or an electrical resonator. Depending on its detuning, the qubit was driven by the resonator either “uphill” (the usual Sisyphus process) or downhill (unusual, or “happy Sisyphus” process). The cycle was completed by relaxation to the ground state in the presence of an additional periodic signal.

Here we demonstrate a new miniaturized clock signal generator based on a memristive (memory resistive) device^17^ operating in a Sisyphus-like cycle. In modern electronics, the clock signal is most frequently produced by quartz generators and sometimes by *RC*-based circuits or other approaches. The quartz generators offer a high precision at the cost of their size. Less precise *RC*-circuits are more compact. Nanoscale memristive devices^18^ [combining their very small size with switching frequencies in a convenient range (e.g., hundreds of MHz)] are the core components of clock generators explored here.

In the literature, there are several known methods of using memristors in oscillating circuits. In particular, significant attention has been focused on nonlinear oscillators constructed from Chua’s oscillators, by replacing Chua’s diodes with memristors^19^,^20^. The authors of ref. 21 proposed a programmable frequency-relaxation oscillator. In such a circuit, a memristor-based digital potentiometer is used to set switching thresholds of a Schmitt trigger. Moreover, memristors can be employed to replace capacitors in relaxation oscillators resulting in compact reactance-less oscillators^22^,^23^,^24^,^25^,^26^. Additionally, it was shown experimentally that some polymeric memory devices exhibit slow current oscillations when subjected to a constant voltage^27^. Our work presents an advanced reactance-less oscillator design having a unique combination of only digital logic gates, a single-supply voltage and a realistic threshold-type memristive device.

In the proposed clock signal generator, the frequency is defined by the switching characteristics of a memristive device. Our circuit employs bi-polar memristive devices^18^ operating in such a way that their memristances (memory resistances) increase/decrease at positive/negative voltages applied to the device, respectively. The effective circuits depicted in Fig. 1(d) show the two stages of its operation. In stage 1 (left circuit), the positive voltage applied to the memristor M causes an increase in its memristance. In the second stage, the device polarity is changed and the memristance decreases. In this way, the memristance oscillates between two values [see Fig. 1(e)], similarly to the oscillations of the boulder height above the ground level in the Sisyphus case [Fig. 1(c)]. Importantly, the circuit does not require any large-size components (such as a quartz resonator) typically used in conventional oscillator circuits.

## Memristive Clock Signal Generator

**Figure 1:**
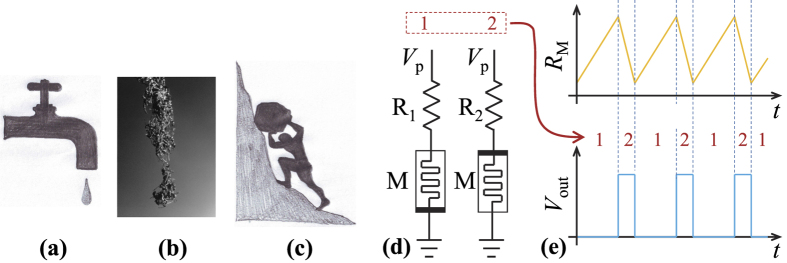
(**a–c**) Examples of Sisyphus cycles: (**a**) leaky faucet, (**b**) dripping ants^7^,^8^, (**c**) mythological Sisyphus. (**d**) Simplified effective circuits realizing a two-phase memristive Sisyphus circuit: the increasing memristance stage 1 (left circuit) and decreasing memristance stage 2 (right circuit). Memristance oscillations and clock pulses are shown schematically on the graphs (**e**). (**b**) is reprinted with permission from ref. 7.

**Figure 2:**
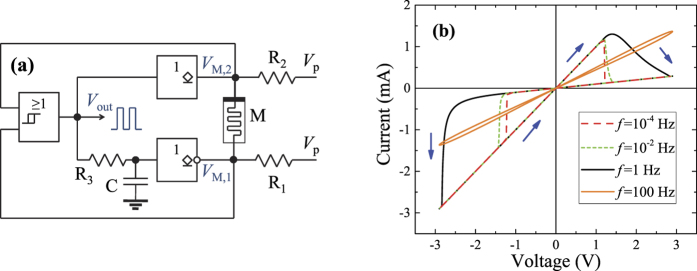
(**a**) A particular realization of a clock signal generator. This circuit employs an OR gate with Schmitt-trigger inputs (to the left), and open-drain identity (upper) and NOT (lower) gates. Here *V*_p_ = 5 V is the power supply voltage. (**b**) *I*–*V* curves of the memristive system M used in this work. The memristive system parameters are given in the text.

Figure 2(a) presents the specific memristive clock generator circuit introduced in this work. Its components include the memristive system M, an OR gate with Schmitt-trigger inputs (to the left), open-drain identity (upper) and NOT (lower) gates, three resistors and a capacitor. Note that the resistors *R*_1_ and *R*_2_ here play the same role as *R*_1_ and *R*_2_ in the effective circuits shown in Fig. 1(e). As will be readily observed, the output of the OR gate defines the operation stages: logical 0 corresponds to the increasing memristance stage 1, while logical 1 corresponds to the decreasing memristance stage 2. The hysteretic input levels of the Schmitt-trigger inputs (denoted by *V*_+_ for the logic 1, and *V*_−_ for the logic 0, *V*_−_ < *V*_+_) are employed as voltage thresholds triggering the stage changes. Figure 2(b) shows the calculated pinched hysteresis loops of the memristive system M described by Eqs (1, 2). In this calculation, *R*_M_(*t* = 0) = 2 kΩ, *V*_M_(*t*) = *V*_0_sin(2*πft*), *V*_0_ = 2.9 V, and all other parameters of M are the same as in the memristor emulator specified below Eqs (1)–(2), 2.

One can notice from Fig. 2(a) that indeed, the increasing memristance stage 1 switches to the decreasing memristance stage 2 as soon as the voltage level *V*_M_ reaches the *V*_+_ threshold. At this instance in time, the output of the OR gate changes to 1, grounding the bottom terminal of M and setting the identity gate into the high impedance state. Assuming that *V*_M,2_ > *V*_−_ right after the switching (below we discuss all the related requirements), the circuit will remain in the decreasing memristance stage 2 while *V*_M,2_ > *V*_−_. The transition from stage 2 to stage 1 occurs in a similar way (as soon as *V*_M,2_ < *V*_−_, and thus both inputs of OR become logical zeros). In Fig. 2(a), the *R*_3_*C* circuit introduces a short time delay to ensure proper switching between the phases. The generator output frequency is not influenced by this delay.

Next, we discuss the experimental implementation of the clock signal generator. In our experiments, the memristive system M is realized with a digital memristor emulator^21^,^28^. Its main parts include a microcontroller, analog-to-digital converter and digital potentiometer. The operation of the digital memristor emulator is straightforward: using the analog-to-digital converter, the microcontroller cyclically measures the voltage applied to the digital potentiometer, calculates an updated value of the memristance (using pre-programmed equations of voltage-controlled or current-controlled memristive system^17^), and writes the updated value of the memristance into the digital potentiometer. Figure 5(b) of ref. 28 presents a photograph of the specific digital memristor emulator realization employed in the present paper. More details regarding the emulator design can be found in refs 29,30.

The memristor emulator was pre-programmed with a model of voltage-controlled memristive systems with threshold^29^,^30^


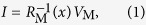



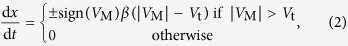


where *I* and *V*_M_ are the current through and the voltage across the memristive system, respectively, and *x* is the internal state variable playing the role of the memristance. Here *R*_M_(*x*) ≡ *x*, *β* is a positive switching constant characterizing the intrinsic rate of memristance change when |*V*_M_| > *V*_t_, *V*_t_ is the threshold voltage, and the + or − sign is selected according to the device connection polarity. Additionally, it is assumed that the memristance is limited to the interval [*R*_on_, *R*_off_] (note that *R*_on_ < *R*_off_). The specific model parameters used in our emulator are *β* = 62 kΩ/V ⋅ s, *V*_t_ = 1.2 V, *R*_on_ = 1 kΩ, and *R*_off_ = 10 kΩ, and *R*_M_(*t* = 0) = (*R*_on_ + *R*_off_)/2. We built the circuit shown in Fig. 2(b) using a TI SN74HC7032 positive-OR gate with Schmitt-trigger inputs, CD74AC05 inverters with open-drain outputs, *R*_1_ = 4.7 kΩ, *R*_2_ = 2.7 kΩ, *R*_3_ = 10 kΩ, and *C* = 1.35 nF.

Our circuit is fully reproducible within the specifications of the circuit components. Assuming that the future memristor devices will be available with well characterized characteristics (similarly, for example, to usual resistors or capacitors), we expect that the suggested circuit based on real memristors will be reproducible too. In addition, our measurements and simulations did not display any significant dispersion of the memristive properties as well of the output signal produced by the circuit. Basically, the memristor emulator operates deterministically and thus the circuit also operates deterministically. As deterministic models are frequently used to describe the response of real memristive devices and, in many cases, show a very good agreement with experiments, we believe that the selected approach (based on the emulator) illustrates our idea in a very realistic manner.

**Figure 3:**
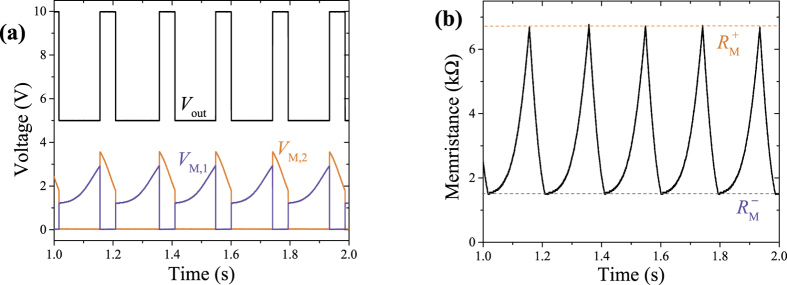
(**a**) Experimentally measured voltages at the output of the OR gate (*V*_out_) and top and bottom electrodes of the memristive system (*V*_M,2(1)_) in the circuit shown in Fig. 2(a). For clarity, the *V*_out_ is displaced by 5 V. (**b**) Memristance *R*_M_ extracted from the data presented in (**a**) by using Eq. (3).

Figure 3(a) presents results of our measurements. While the *V*_M,1_ and *V*_M,2_ curves clearly demonstrate two stages of the circuit operation, the output *V*_out_ [displayed in Fig. 2(a)] shows a stable clock signal with a period of about 0.194 s and a duty cycle of about 27%. With the knowledge of *R*_1_ and *R*_2_, the *V*_M,1_ and *V*_M,2_ curves were used to extract the time dependence of the memristance *R*_M_ depicted in Fig. 3(b). Its variations are similar to the desired variations of *R*_M_ sketched in Fig. 1(e).

## Circuit Analysis

In Fig. 3(b), the memristance *R*_M_ of M changes periodically from 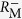 to 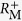, and back (see also Fig. 1(e)). Analytically, one can find the corresponding durations of the time intervals, *τ*_1_ and *τ*_2_. In what follows, we focus on the effective circuit models depicted in Fig. 2(d) to find *τ*_1_ and *τ*_2_ analytically.

For the sake of convenience, let us consider the system dynamics from *t* = 0 in both stages and perform calculations based on the memristive device model employed in our emulator [Eqs (1, 2)]. Then, using


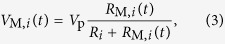


where *i* = 1, 2 denotes the two time intervals, we integrate Eq. (2) with appropriate initial and final conditions for both intervals, and accounting for the memristor polarity. As a result, one can find the following expression for the duration of the two stages


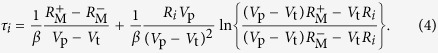


The oscillation period is given by


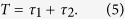


The boundary values of the memristance, 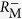 and 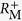, can be expressed through the Schmitt-trigger input thresholds as


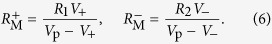


The expression for the period *T* can be written in a simple form assuming that *R*_1_ = *R*_2_ and *V*_p_ ≫ *V*_t_ (and also 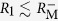 so that 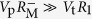). Then we obtain


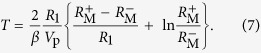


This together with Eq. (6) gives


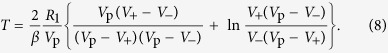


**Figure 4:**
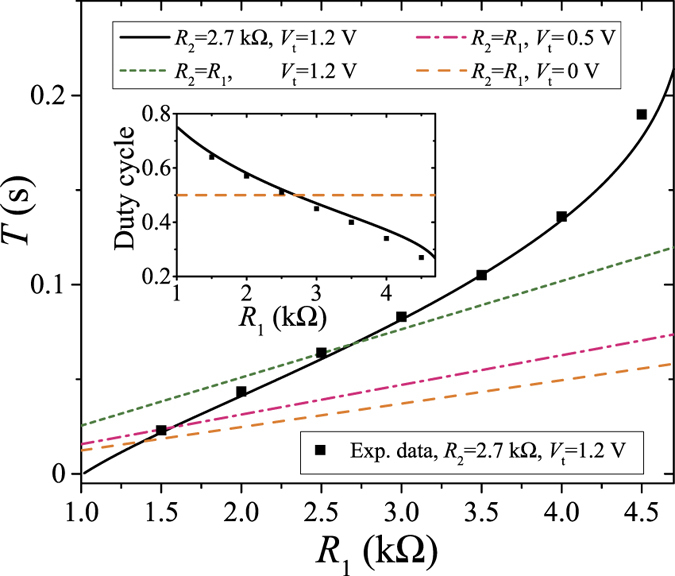
Period *T* = *τ*_1_ + *τ*_2_ as function of the resistance *R*_1_. Inset: the duty cycle *τ*_2_/*T* as a function of *R*_1_. This plot was obtained using the following set of parameters: *V*_p_ = 5 V, *V*_+_ = 3 V, *V*_−_ = 1.8 V, and *β* = 62 kΩ/V ⋅ s. Note that *R*_2_ and *V*_t_ are indicated on the plot.

The above formulas are plotted in Fig. 4. There, it is assumed that all the circuit parameters (except *R*_1_ and *R*_2_) are fixed. This figure shows that both the period *T* and the duty cycle *τ*_2_/*T* can be tuned in a certain range by varying *R*_1_ and/or *R*_2_. In Fig. 4, the solid curve was plotted for the same value of *R*_2_ as used in the experiment, while the other curves demonstrate the changes assuming equal resistances, *R*_2_ = *R*_1_, for several values of the memristor threshold voltage *V*_t_. In particular, Fig. 4 demonstrates that the formula (8), which is valid at *V*_t_ = 0 and *R*_2_ = *R*_1_, provides a good estimation for the period *T* for small values of the threshold voltage *V*_t_ when *R*_2_ = *R*_1_. Moreover, Fig. 4 also shows several experimentally measured periods and corresponding duty cycles that exhibit a very good agreement with our analytical results.

Finally, we consider the limitations imposed on the clock signal generator components required for its proper operation. The numerical estimations provided below employ parameter values that are close to those of our experimental implementation of the signal generator. For the convenience of readers, here we list the values of these parameters: *V*_p_ = 5 V, *V*_+_ = 3 V, *V*_−_ = 1.8 V, *V*_t_ = 1.2 V, *R*_on_ = 1 kΩ, and *R*_off_ = 10 kΩ.

1. In the stage 1, the minimum (maximum) values of *V*_M_ must be below (above) *V*_+_; namely, *V*_M_(*R*_on_) < *V*_+_ and *V*_M_(*R*_off_) > *V*_+_. Consequently,


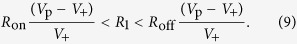


Numerically, 0.667 kΩ < *R*_1_ < 6.67 kΩ.

2. In the stage 2, the minimum (maximum) values of *V*_M_ must be below (above) *V*_−_, namely, *V*_M_(*R*_on_) < *V*_−_ and *V*_M_(*R*_off_) > *V*_−_. Consequently,


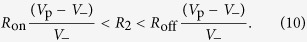


Numerically, 1.778 kΩ < *R*_2_ < 17.78 kΩ.

3. Moreover, right after the switching from stage 1 to stage 2, the voltage across M should stay above *V*_−_, namely, 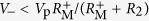, where 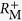 is given by Eq. (6). It follows that


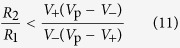


and, numerically, *R*_2_/*R*_1_ < 2.67. We note that the requirement that in the transition from stage 2 to stage 1 the voltage across M stays below *V*_+_, is also given by Eq. (11). For the same transition, one can also require that 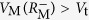 in stage 1, however, this requirement is weaker than the criterion 4.

4. In order to start oscillations from *any* initial condition, the voltage across M (in the worst-case limit *R*_M_ = *R*_on_) should exceed *V*_t_. This results in


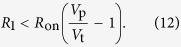


Using the above-mentioned parameters, we obtain *R*_1_ < 3.167 kΩ.

5. Lastly, we mention the obvious requirement on *V*_−_, namely, *V*_t_ < *V*_−_.

One can easily notice that in our experimental implementation of the circuit shown in Fig. 2(b), the selected values of *R*_1_ = 4.7 kΩ and *R*_2_ = 2.7 kΩ satisfy the criteria in Eqs (9, 10, 11). Regarding the criterion (12), we were able to use a large value of *R*_1_, because the initial value of the emulator memristance was selected above *R*_on_.

## Conclusion

We have proposed and analyzed the design of a memristive clock signal generator. Assuming a realistic threshold model of a memristive device, we experimentally demonstrated the operation of a memristive frequency generator. While our demonstration is based on a slow memristor emulator, the real memristive devices can result in frequencies in the industrially important MHz-GHz range. Moreover, the specific proposed circuit for frequency generation offers frequency and duty cycle tunability. These theoretical considerations together with our experimental emulation shows the potential of such circuits for very compact frequency generators.

## Additional Information

**How to cite this article**: Pershin, Y. V. *et al*. Memristive Sisyphus circuit for clock signal generation. *Sci. Rep*. **6**, 26155; doi: 10.1038/srep26155 (2016).
